# Pathology, Practical Medicine, and Therapeutics

**Published:** 1838-04

**Authors:** 


### PATHOLOGY, PRACTICAL MEDICINE, AND THERAPEUTICS.

### *Notice of the Influenza which prevailed in Lisbon during the month of February*, 1837. By Dr. Lima Leitao.

The labour of comparing the character and progress of epidemics in climates very different from our own with what we observe of their essential nature and mode of transmission at home is one of considerable interest, and may, we consider, prove instrumental in illuminating this one of many obscure departments of our art. Dr. Leitao's memoir bears on the face of it the impress of faithfulness and candour, and is a very detailed and ample account of the disease; but in our eyes its value consists chiefly in the differences we discover not only in the character of the epidemic in Portugal and in Britain but in the period of its appearance, the weather with which it was associated, and certain circumstances connected with its diffusion in the respective countries. The influenza was milder in its character in Portugal than here, evinced by the fact of its not being fatal in the former situation. Besides this difference, we find, according to our author, that gastric symptoms, when they occurred in Portugal, were the results of treatment, emetics, strong purgatives, or stimulating diaphoretics, having been administered, and having given origin to them, whereas we can assert from our own abundant observation that in this country, gastric symptoms, liable certainly to be aggravated by improper treatment, constituted an important portion of the majority of cases observed in the epidemic—in many instances, indeed, a more important portion than the affection of the mucous membrane of the air-passages.

The winter in Lisbon was not unusually severe, but the early days of January were very cold, so much so that on the 2d of that month snow fell so abundantly as to give the Portuguese capital the appearance of a city in the north of Europe, and astonished the inhabitants very much, nothing similar being remembered. But from the 10th of February to the end of the same month, the period during which the epidemic appeared, the sun was very warm though the wind was high and cold. The disease, it will be observed, manifested itself fully six weeks later than in this country. We at least saw cases towards the close of December, 1836, whilst the first examples that fell under the observation of Dr. Leitao were on the 20th of February. On the first appearance of the epidemic in England the weather was so severe, as to be very generally supposed the cause of the disease, but in Portugal the severe weather engendered no epidemic, but the influenza there, like our own of 1833, appeared and spread under a warm sun. The author informs us that it was rumoured that some English ships, recently arrived from England and lying in the Tagus in the beginning of February, had influenza on board, and to this circumstance he is evidently disposed to ascribe its diffusion through the city. It originated, he says, under the same circumstances at Bilboa in the north of Spain, there being English ships there. He regards the cause of the disease atmospheric, and thinks that fractional portions of the morbific atmosphere may be conveyed in ships and become the cause of contamination to an atmosphere hitherto pure. We remember that the late Sir David Barry propounded a similar theory clothed in very mystic terms respecting the transmission of cholera. It appears to us at most only a circumlocutory expression for a mode of contagion very well exemplified in the case of puerperal fever, where a medical man conveys from bedside to bedside a disease from which he is himself exempt. Dr. Leitao thinks that influenza in its diffusion through Portugal manifested a preference for the courses of rivers; and states that ascending the Tagus *especially by the right bank*, it reached Abrantes and Villa Velha. This preference for the *right bank* of this great inlet to the interior of central Portugal must certainly be referrible to some other principle than a tendency to move in the course of a river, and, as we happen to know that the halting places of the barges navigating the Tagus are chiefly on its right bank, supposing the fact to be correctly stated by the author, we cannot explain it otherwise than by considering human intercourse to have been the means of diffusing the disease. This statement of Dr. Leitao bears more the aspect of contagion than anything that fell under our observation in this country.

*Jornal da Sociedade das Sciencias Med. de Lisboa. Jan.* 1837.

---

*The Influenza in Copenhagen in the winter of* 1836-7. By Dr. OTTO, Professor in the University of Copenhagen.

THE first cases took place in December, 1836, simultaneously with the occurrence of the epidemic at St. Petersburg, Stockholm, Berlin, &c., but a year later than in most other parts of Europe. Former visitations of the disease had taken place in spring. The influenza was ushered in, and accompanied in its progress, by the same thick foggy weather which had introduced the measles the year before; it spread with astonishing rapidity; family after family was attacked in rapid succession, and when the epidemic was at its height, in the middle of January, upwards of 30,000 persons were ill at one time. Towards the end of January the disease began to decline, but continued through the month of February, and even at the beginning of March some slight cases occurred. The symptoms were those of catarrh with or without fever, but invariably accompanied by extreme depression of strength, the marked attendant of every former epidemic; a dry cough, with or without pain in the chest, hoarseness, great thirst, loss of appetite, furred tongue, nausea, bitter taste in the mouth, and headach were present in a greater or less degree. When the patients attempted to follow their usual avocations the symptoms were aggravated, and the disease often lasted as long as a fortnight, whilst rest and careful management removed every symptom but weakness in four or five days. Perfect health and strength, however, were rarely restored under five or six weeks. Exposure to cold soon brought back the disease in an aggravated form. The prognosis was nearly always favorable, for the epidemic was distinguished from those of London, Glasgow, &c., by being in itself entirely free from danger. Persons affected with cough or diseases of the lungs, and old men suffered most, and children least. Dr. Otto concludes his memoir by stating his firm conviction that the influenza at Copenhagen was contagious, and that when one member of a family was attacked the others were sure to suffer in succession.

*Zeitschrift fur die gesammte Medicin.* B. v., Heft ii. *Hamburg, June* 1837.

---

*Method of treating Intermittent Fevers in the Infirmary of Clinical Medicine of the Surgical School of Lisbon.* By LIMA LEITAO, M.D., Professor.

[AGUE being of very frequent occurrence among the labourers in the flooded or marshy grounds bordering on the Tagus, Dr. Leitao has written a sensible article explanatory of the varieties observed in the disease, and of the treatment applicable to each variety. It appearing to us to be a very judicious account of the abdominal complications of recent intermittents, and to be faithfully deduced from ample observation, we transfer it in an abridged form to our pages. Such of our readers as are not, like the author, disciples of Broussais, will probably regard the local affections of the abdominal organs as concomitants not as causes. We shall, however, retain the original divisions and designations.]

The following divisions comprise the varieties observed: 1st, intermittents proceeding from gastro-duodenal phlogosis; 2d, those arising from inflammation of the liver, of the spleen, or both conjointly; 3d, such as proceed from phlogosis embracing simultaneously, wholly or in part, the gastro-duodenal mucous lining, the liver, and the spleen; 4th, intermittents not arising from inflammation.

1. The symptoms of the cases referred to the first division are thus described. If during the intermission, the following phenomena are observed—redness of the margin of the tongue, with a white or slightly yellow coating on its surface; more or less thirst; an obscure feeling of pain or weight in the epigastrium, even when pressure is not applied; a sense of heat in the urethra and rectum in passing urine and fæces; nausea, or vomiting of mucous or bilious matter; a pulse without being decidedly febrile, yet not that of health; then, according to the author, there exists inflammation of the mucous membrane of the stomach, duodenum, or both. This form of the disease Dr. L. has observed exclusively in persons of the sanguineous, or bilio-sanguineous temperament; of youthful and adult age; of a constitution not yet broken-down, and in first attacks of ague. He does not remember to have observed it in the quartans of Portugal; but only in quotidians, and double and single tertians. For its cure he recommends, repose in bed; a diet of light broth; beverage lightly acid and edulcorated (*agridoce*) or mucilaginous, according to the taste of the patient, taken tepid; and emollient *enemata*. After the second paroxysm, or after twenty-four hours' repose in the hospital, whatsoever number of paroxysms besides the second may have occurred prior to the patient's admission there, leeches are applied to the epigastrium, followed by poultices. The number of leeches is proportioned to the age of the patient, twenty-four being the mean number. Dr. L. thinks the paroxysm the most suitable period for their application. Should the symptoms above described have disappeared in the apyrexia next ensuing, but should the paroxysm follow with the same or nearly the same intensity, a grain of the sulphate of quinine is given every three hours, every two hours or every hour (according to the type of the disease) during the intermission. After each dose of the medicine Dr. L. gives some mild mucilaginous or sugared beverage, and very little other sustenance is taken. It is important that the apyrexia be perfect, and that the indications of local affection have ceased before administering the sulphate of quinine, otherwise there is risk of converting the disease into the remittent or continued form.*

Should the inflammation resist the first application of leeches, they must be repeated a second or even a third time, till it is removed. If the paroxysm does not recur after the application of the leeches, or if it be much diminished in intensity and later in coming on, no sulphate of quinine is administered; and, in the latter case, it is observed, that after one or two fits more and more slight, the disease ceases. The author thinks that convalescence is more speedy and relapse less liable to take place in these, as it were, spontaneous recoveries, than where the sulphate is employed.

2. In the second division, comprising cases in which the liver, the spleen, or both conjointly are affected, the author recognizes the phlogosis of the liver (should there not be enlargement) by obtuse pain, heat, and tension, increased by pressure; yellow tinge of the face and eye; yellowish furred tongue; bilious vomiting and dejections, &c. When the liver is enlarged, the local symptoms are referred to the left lobe. If the spleen suffers, it presents analogous local symptoms: both organs are often simultaneously affected. The remedies of this form of the disease are the same as those of the preceding, with this difference that general bloodletting is found more serviceable than leeches, or, at least, should precede their employment. Two bleedings of eight ounces each are generally sufficient. Dr. L. has seen intermittents of this division and of all types, yield to depletion alone by general followed by local bloodletting; and this successful result from depletion solely has been more manifest in the diseases of this than the preceding division.

3. The third division, comprising intermittents connected with inflammation of the gastro-duodenal lining, and of the liver and spleen conjointly, is marked by a combination of the symptoms of each of the preceding diseases. The malignant intermittents, observed by the author in eastern Africa, belonged to this class. The treatment consists of the methods employed for the other two divisions combined, that is, bleeding, general and local, excepting in nervous temperaments, when he has recourse only to the latter. He thinks this kind of case very suitable for the endermic method of employing sulphate of quinine. In the malignant

* This admonition we think of great importance, having seen very serious evil result from the neglect of such caution.—REV.

intermittents of eastern Africa, he derived much advantage from frictions of tincture of bark, and from sprinkling blistered surfaces with powdered bark and camphor.

4. The fourth division, consisting of cases unattended with local inflammation, he treats as he does those of the preceding, except that bleeding is omitted.

The author subsequently gives a practical commentary on the 59th aphorism of the 4th section of Hippocrates, "tertiana exacta in septem circuitibus ad summum judicatur." Having tried its truth, he found the patient, solely from the influence of low diet and repose, escape the seventh paroxysm in some cases and the eighth in others. The examples in which this fortunate result took place belonged principally to his fourth division; but a proportion of them to his first, or that comprising the complications with gatro-duodenal inflammation. These spontaneous recoveries, wheresoever they occur, Dr. Leitao regards as the most favorable, the general health being the least disturbed, convalescence most prompt, and relapse very rare.

[We recommend these "spontaneous cures" of ague, to the attention of Mr. Parkin, (See British and Foreign Review, No. V. p. 199,) as throwing some light on the result of his treatment of the same disease in Spain, by means of saline draughts.]

*Jornal da Sociedade das Sciencias Medicas de Lisboa.*
*Mez de Fevereiro, e de Abril,* 1836.

---

### *Observations on the Plague.* By Dr. Bulard.

Dr. Bulard has enjoyed opportunities of studying the fatal malady in several of the cities of Egypt, of Asia Minor, and Turkey; and the following remarks are abridged from some abstracts of his views communicated to the *Medicinische Zeitung*, by Dr. Vetter. Dr. Bulard is now in Egypt.

After mature consideration, Dr. Bulard adopts the hypothesis that the plague depends upon alteration of the fluids. When a vein is opened, the blood escapes freely, but it is thicker, and its colour is darker, than in the healthy condition. After standing for some time, it acquires a violet hue, and forms an imperfect coagulum, covered with a deep reddish coloured serum. Frequently, however, no coagulum is formed: the blood in that case becomes of a livid hue, has a very perceptible odour, and some oily drops may be observed upon its surface. After death, the blood in the arteries and veins is found equally dark-coloured; and in some of the large trunks the oily drops are also occasionally discovered. These appearances, which the blood presents after death, are not the result of post-mortem changes; for the period which elapsed between death and the examination of the body was insufficient to produce this by decomposition. The first link in the chain of disease is in the lymphatic system: a diseased lymph is poured into the blood, and gives rise to all the fearful phenomena of plague, by acting as a poison in a manner analogous to the action of pus in phlebitis, according to theory of Dance. The alterations in the tissues of the body are such as might be expected from alteration of the fluids. The spleen, in ninety-five cases of a hundred, is more or less diseased; the stomach, in about twenty cases of a hundred, presents diseased appearances; but these do not appear to stand in any necessary relation to plague. The liver is occasionally slightly altered in colour and density; the lungs and bronchial mucous membrane are sometimes inflamed, but are as often healthy; and the only constant, and apparently the most material, lesion which occurs is venous congestion of all the tissues of the body,—of the brain, lungs, liver, spleen, heart, kidneys, &c.

The disease may be divided into two periods: the symptoms of the first are an increased development, in a greater or less degree, of the lymphatic glands, with softening or putrid degeneration of their substance, and venous congestion of the great vessels. The second period is characterized by a putrid state of the lymphatic glands, with extravasation of blood; general softening of the tissues; a decided state of general congestion; external and internal petechiæ; ulceration of the gastric mucous membrane; ecchymoses, and carbuncles.

Resting upon these views, various methods of treatment were assayed; but the result was seldom satisfactory. At first, patients who presented the characteristic symptoms of the first period, (in general, from about six to twelve hours after the commencement of the disease,) were treated with stimulant and volatile remedies; such as muriate and acetate of ammonia, volatile ammonia, iodine, chlorine, nitric acid, and the preparations of alcohol and æther; but these remedies produced no perceptible effect upon the nervous system, although they sometimes recalled the secretions of the skin and kidneys, and produced a momentary increase of muscular strength. The patients thus treated generally died in from twenty-four to forty-eight hours. Cases of the same description were next treated by emetics: these produced vomiting and perspiration, but had no beneficial effect upon the course of the disease. Narcotics, although in opposition to theory, were next tried with a like result. Sulphate of quinine, calomel, pills of mercurial ointment, combined with external mercurial frictions, had no effect in altering the character of the disease.

The best mode of practice seemed to be that which was limited to assisting nature, and such a method was therefore ultimately adopted. The feeling of cold was combated by additional blankets and the exhibition of warm drinks; the buboes were brought to a head by poultices, and laid open as soon as fluctuation was distinct; carbuncles, when present, were punctured, or, when they did not appear within a certain time, were artificially produced; and, in short, the footsteps of nature were followed as closely as possible.

Numerous experiments have been instituted to determine precisely the nature of the contagious principle of plague, but still all doubt upon the subject has not been removed. It has, indeed, been fully proved that immediate contact suffices to spread the disease; but, notwithstanding the experiments of the commission of Cairo, it still remains doubtful whether mediate contact possesses a like power. The experiments of the commission were performed in the plague-hospital; and hence the doubt has arisen whether the disease, in their experiments, did not arise from atmospheric or other causes, independent of the operation of mediate contact.

*Experiment* 1. On the 15th of May, 1835, Dr. Bulard stripped himself in the wards of the plague-hospital, and put on the still-warm shirt of a patient ill of plague, and continued to wear it for forty-eight hours, without adopting any precautionary measures to prevent absorption. No evil consequences followed.

*Exp.* 2. On the 17th Zilkedjè, at eight o'clock in the evening, the condemned criminal, Ibrahim Hassan, æt. 18, was dressed in the shirt, jacket, and trousers, and put into the still warm bed of a patient ill of plague: on the evening of the 21st symptoms of plague appeared, and he died in the night of the 25th.

*Exp.* 3. At eight o'clock of the evening of the 7th Zilkedjè, the condemned criminal, Mohamed-Ebn-Ali, was dressed and put to bed in a similar fashion. He continued in his usual health till the morning of the 23d, when the disease appeared, and on the 26th he was convalescent.

The inoculation of dogs with the pus of buboes, with blood from the veins and heart, or with the serum of carbuncles, produced no consequences; neither had the introduction of the serum of carbuncles into the subcutaneous cellular tissues of dogs and asses any effect which could be ascribed to an animal poison. Dogs which devoured these pathological productions likewise continued healthy.

*Exp.* 4. On the afternoon of the 18th Zilkedjè, the condemned criminal, Hassan, was inoculated in four places in the right arm with blood from the cephalic vein of a plague patient. He was seized with plague on the 20th, and was convalescent on the 25th.

*Exp.* 5. On the 22d Zilkedjè, the condemned criminal, Mahomed Halil, a plethoric robust man, was inoculated with the blood of a plague patient; and again on the 30th, after an interval of eight days. The punctures, owing to mechanical irritation, became slightly inflamed, but no general symptoms followed.

The same man was afterwards inoculated with pus from a bubo; and Mohamed-Ebn-Ali, who had now recovered from the effects of the former experiment upon him, was inoculated with serum from a carbuncle: the disease did not appear in either case. Clot-Bey now inoculated himself in the axilla and groin, with a like negative result.

In those cases mentioned above, where the disease appeared to be produced by mediate contact, it remained doubtful whether this was really the case, owing to the circumstances in which the experiments were performed; and the same causes invalidated the conclusions which might have been drawn from those cases in which the inoculation took effect.

From the fatal nature of the disease, it should be combated chiefly by prophylactic measures; and these Dr. Bulard considers under six heads: 1, the preparations of iodine; 2, the preparations of mercury; 3, frictions with oil; 4, inoculation with the matter of buboes and carbuncles; 5, inoculation with vaccine matter; 6, epispastics. Nothing as yet is known as to the preservative power of the two first; popular opinion speaks for the third. It has been asserted that small-pox acts as a preservative; but this still remains a doubtful question, although in the Levant the voice of the majority has pronounced it as such. Epispastics are rarely found on the bodies of those who have died of plague; and it is said that, of forty thousand cases which died in Constantinople, there was not one body on which was found an open issue; and Dr. Bulard affirms that, of fifteen hundred cases admitted into the hospital of Cairo, on one only an issue was found. From these facts it has been asserted that individuals with issues enjoy almost total immunity from the attacks of the disease.

*Medicinische Zeitung,* 1837. Nos. 40, 41, 42.

---

*On the Medicinal Powers of the Caïnca.* By Dr. ROBREDO.

BARON LANGDORF, Russian consul-general at Rio Janeiro, first announced, a few years ago, the wonderful virtues of this plant in Europe. It is the *Chiococca racemosa* of Linnæus, and of the natural family *Rubiaceæ* of Jussieu, and is very abundant in the Antilles, the Floridas, and many parts of Brazil. Its root is composed of a delicate bark, which has a bitter and slightly acrid and astringent taste, and, when broken, offering a resinous aspect; and a woody centre, which does not partake of these properties. Messrs. Pelletier and Caventou have found that it contains a bitter principle, to which they have given the name of Caïncic acid, an oily matter of a green colour, another of a yellow colour, and a coloured viscous substance. From the experiments of Messrs. Caventou and François, it appears that the root, and especially the caïncic acid, are powerfully tonic; that they increase the secretion of urine in a very remarkable manner, and that they are slightly purgative.

Three cases are related by Dr. Robredo in confirmation of these opinions. In the first case, a bilious diarrhœa had reduced the strength of the patient, a female, extremely; the suppression of this diarrhœa being always followed by thirst, scanty urine, extensive œdema, and excessive prostration. A recurrence of the diarrhœa removed these symptoms, but exhausted rapidly the strength of the patient. The employment of caïnca was begun, when the dropsical symptoms were extreme, and the urine scanty and sedimentous. A decoction of a drachm of the root in a pint of water was administered daily. The second day of its employment ten pints of clear urine were discharged, the stomach performing its functions regularly; and in five days a cure was effected, which the author thinks is radical, since, at the period of his writing, three months had elapsed without any disturbance of the patient's health, and her strength was quite restored.

In the second case, amenorrhœa had been treated by copious bloodletting, and anasarca was the consequence. The author having ascertained, by examination, that there was no disease of the uterus, administered caïnca. The ultimate effect was more slowly produced; fifteen days having been required for a cure, and the dose of the medicine (probably from deficient susceptibility of the patient,) having required increasing to a drachm and a half daily.

In the third case, that of a boy eight years old, there was extreme marasmus, œdema, costive bowels, and enlargement of the spleen; the whole the result of an ague of three months' duration. All the symptoms, the enlargement of the spleen excepted, were removed in eight days, by means of a decoction of half a drachm of the root daily; and, by appropriate remedies, the spleen was afterwards reduced to its natural condition.

*Jornal de la Academia de Medicina de Megico. Octubre,* 1836.

---

*Notice of the Bafureira of Cape de Verd.* By Dr. B. A. Gomes.

This plant is indigenous at Cape Verd, and is employed by the inhabitants of the Cape Verd Islands and the coast of Africa to increase the secretion of milk. The writer has been assured by persons whom he thinks worthy of credit, that not only is it efficacious for this purpose in women who have been recently confined, but that it produces the secretion in virgins and persons of advanced life; so that infants have been nursed for a long time by females who, from their age and other circumstances, could not have furnished the secretion under the influence of any natural stimulant. Its mode of employment is by means of poultices, made of the green leaves, applied to the mammæ; or a strong decoction, with which the same parts and external organs of generation are washed. Sometimes such a decoction is taken internally, conjointly with its external application. The plant belongs to the family *Euphorbiaceæ* and the genus *Ricinus*. The writer is doubtful whether it is a variety of the *Ricinus communis*, or a distinct species. It has been raised in the garden of the Marine Hospital at Lisbon, from seeds sent from Cape Verd.

*Jornal da Sociedade das Sciencias Medicas de Lisboa. Novembro,* 1836.

---

*Researches on the Febrifuge Properties of the Chloride of the Oxide of Sodium.* By Dr. Gouzée.

The high price of the sulphate of quinine renders it very desirable that some other remedy, more attainable by all classes of society, should be discovered for the effectual treatment of intermittent fevers. In a memoir presented to the Academy of Sciences by Dr. Lalesque, in 1835, the chloride of soda was recommended as possessing medicinal properties as active as those of the sulphate of quinine, and as being fitted to serve as its substitute in all cases in which the quinine is indicated in periodic fevers. Dr. Gouzée gave, at first, but little credit to the value attached to the chloride, until it was further confirmed by the experience of a friend; but, to establish the efficacy of a new febrifuge remedy, various conditions are indispensable. It is not only necessary that the situation generally imparts to intermittent fevers that tenacity which prevents their yielding to all medicines: attention must also be paid to the particular case in which the experiment is made, to the season, to the medical constitution; which circumstances will alone, in certain circumstances, contribute powerfully to recovery. Thus, for example, it has just happened that at Antwerp, where the most favorable circumstances might appear to be combined for experimenting on this chloride, a number of fever patients have become spontaneously convalescent, shortly after their entrance into the hospital; having suddenly passed from a life of activity, and an abundant but not well selected diet, to circumstances of an opposite character. It is also very important to determine the quality of the medicine employed, as well as its exact mode of administration. In these matters too much exactness cannot be observed. The chloride of soda employed by Dr. Gouzée was recently prepared, marking 12 of the areometer, and decolorizing at least eight parts of the sulphate of indigo. The ordinary prescription has been half a drachm of the chloride in four ounces of distilled water. The patients have so taken this dose that the last quantity of it should be swallowed shortly before the occurrence of the paroxysm which it was wished to overcome; and, in order to isolate the patients as much as possible from all opposing influences, a very light diet only was allowed, and confinement either to the bed or chamber was enjoined.

Several cases are recorded, illustrative of the febrifuge action of the chloride of the oxide of sodium, and from them the following inferences appear to be fairly deducible:

1. The chloride of soda actually possesses febrifuge properties. 2. It is far from producing the certain and energetic effects of the sulphate of quinine. 3. It cannot, therefore, replace the sulphate of quinine in every case in which that salt is indicated in intermittent fevers; and it would be imprudent to hazard its employment in pernicious intermittents. 4. It is not irritant. 5. It may be had recourse to in recent intermittent fevers, disposed to yield, in individuals who are easily impressed, in women, in children; and it may, in general, be employed in all cases where there does not exist any danger. 6. The diminution of the intensity of the paroxysms during its use augurs favorably, but does not always announce an approaching cure. 7. It appears to exercise a favorable influence over engorgements of the spleen. 8. It remains a subject for further enquiry whether its dose and mode of administration may not be advantageously modified; if it may not be associated with other substances capable of rendering its action more energetic; if, lastly, in continuing its use, the frequency of relapse may not be diminished.

*Revue Médicale française et étrangère. Février*, 1836.

---

*On Obliteration of the large Arteries.* By M. Legroux.

At the sitting of the Academy of Medicine, on the 21st February, M. Legroux related the case of a lady, aged twenty-nine, who, three months previously, was seized with acute rheumatism. There was uneasiness in the præcordial region, an irregular pulse, and a loud bellows' sound, and these symptoms were attributed to endocarditis. Antiphlogistic treatment was employed. At the end of a month she appeared to be getting well, when all at once violent pains came on in the legs and feet, with coldness of the extremities. In a fortnight the arterial pulsations had completely ceased in the lower limbs. Eight days before death, the same symptoms were observed in the left arm, and the pulsations ceased there also. The pains continued to the last; no gangrene took place.

On inspection, the cavities of the heart were found to contain old and adherent fibrinous clots; the lining membrane of the auricles had lost its polish and transparency; the right subclavian, the termination of the aorta, the common and external iliacs, completely obliterated by old and adherent clots; the right iliac dilated at the level of the hypogastric to the volume of a nut, and containing a fibrinous, cystiform coagulum; three of the lumbar arteries were closed by clots, which projected into the aorta like the heads of nails.

M. Legroux thinks, 1st, that this disease was, in the first instance, rheumatic endocarditis; 2d, that, in consequence of this inflammation, coagula were formed in the cavities of the heart; 3d, that the obliteration of the arteries was commenced by the expulsion of coagula from the heart into the vessels. This opinion he founds on numerous observations, which prove the very frequent coincidence of coagula closing the arteries and old clots in the heart: he, however, admits that local obliteration may be caused by arteritis. 4th. That the obliteration, at first imperfect, was completed by successive additions of coagulum; the structure of the clots indicating that they had been formed at various times. 5th. That the adherence of the coagula to the walls of the artery was the result of inflammation caused by the presence of the clot as a foreign body. The inner membrane of the artery was only notably altered at points where the obliteration had existed for some time; that of the subclavian, which was recently closed, was only slightly injected.

*Bulletin de l'Academie.* 15*th March*, 1837.

---

*On a particular Kind of Swelling of the Tonsils, Uvula, and soft Palate.* By Dr. Rösch.

In obstructions of the portal system, in deficient activity of the mucous membrane of the intestinal canal, and particularly in diseased states depending on sluggishness of the intestinal secretions, the tonsils, uvula, and velum swell from time to time, still remaining soft: they appear to be traversed by small blue vessels, and to have acquired a blueish dark-red colour; the swallowing is somewhat impeded, and the voice not unfrequently acquires a hoarse tone. This condition may be apparent one day, and lost on the following; but it frequently continues many weeks, and disappears only on recovery. In general it keeps pace with the improvement or deterioration of the general complaint, so that, e. g., on the re-establishment of the action of the bowels by means of laxatives, it disappears. The author considers such a condition of the throat always as a symptom of obstruction, and also as a symptom of chronic gastritis, in cases where the patients are already very much debilitated, where there is an evident predominance of the venous system, and where there is a tendency of the blood, however small, to be evacuated by the intestinal canal. An analogous state of parts is seen in the blue discoloration of the lower lip in hydrothorax, hemorrhoids, &c., and which is indicative of obstruction.

*Jahrbücher der in-und-auslandischen gesammten Medicin.* No. 2. Heft 2. 1837.

---

*Case of Chronic Hydrocephalus, cured by accidental Paracentesis.*
By Dr. Höfling, of Heinfeld.

The patient, a boy, æt. 5, presented the symptoms of chronic hydrocephalus in a marked degree. The temporal and frontal regions of the head were, in particular, largely developed, and projected far over the diminutive face. The general health, however, still continued good, and there was no particular ailment. Whilst in this state, the boy received a severe blow on the forehead from the hoof of a cow, which produced momentary stupefaction. Examination shewed that the frontal bone, already extremely thin, had been broken by the stroke, and that a considerable quantity of water had already escaped, and still continued to flow from the wound. The water continued to come away in a steady flow during the eight following days, when the wound closed. Two years have now elapsed since this accident, and the boy is in the enjoyment of good health. The head is still large when compared with the face, but its proportion to the body is nearly natural.

*Wochenschrift für die gesammte Heilkunde.* 1837. No. 41.

---

*Practical Observations on the Treatment of Diseases with Milk and Whey.*
By Dr. Leviseur.

Dr. Leviseur's experience of the utility of milk and whey is confined to a few diseases: on the different value of different kinds of milk, he has not made any experiments, that of the cow being the milk which he almost always employed. We shall notice his remarks on the utility of milk in some chronic diseases; and of its utility, as a remedy of more value than any other with which he is acquainted, in these diseases, he speaks almost enthusiastically. His patients were children and young people under thirty years of age. The common character of all the diseases was emaciation. 1. Children in their second year, not long after their removal from the mother's breast, and before the end of teething, sometimes become the subjects of diarrhœa, which, from neglect or mismanagement, acquires great obstinacy, assumes the character of lientery, is attended with increasing emaciation, and terminates life, with or without dropsy. They are emaciated and debilitated, the skin hanging in loose folds about them; they swallow what is offered them to eat, and void it, per anum, almost immediately, mixed with a peculiarly fetid mucus. The collapsed and soft belly particularly distinguishes this condition from the so-called atrophy of children. This is a condition in which Dr. L. has especially employed the milk-system, and with constant success; provided always that no *febricula hydrencephalica*, no deep-seated organic lesion, or no dropsy exist, and that the treatment is pursued strictly according to the prescribed rule.

*Mode of Treatment.* Medicine is to be totally excluded. If a good nurse can be procured and the child will submit to it, it may be put again to the breast; but this cannot often be managed. Cow's or goat's milk, somewhat diluted with water but not sugared, either just milked or boiled, must be given to the child *without*

*limit: let it drink as much as it may.* It is taken with great appetite, and in such abundance as to swell up the belly; the lookers-on being apt to say that the child must burst. Sleep generally follows; the urine flows in considerable quantity, and the relaxed evacuations from the bowels appear to consist of nothing but coagulated milk. Nothing whatever but the milk is to be given to the child. It may be repeated in the same manner, morning, noon, and evening. If the child has been indulged with various kinds of food previous to this treatment, it may require firmness on the part of the parent and nurse to refuse that for which it will cry; but in two or three days, the child will contentedly see others eat, itself being perfectly contented with its milk. In the course of from eight to fourteen days, the improvement becomes evident, the diarrhœa diminishes in frequency, &c. Before eight weeks have elapsed, the lientery has long ago ceased, the condition of the child is wonderfully improved, and in general several teeth will have protruded themselves. If at this time some other diet is had recourse to, its chief ingredient should be milk, and this until the first dentition is ended. Errors in diet and cold readily bring back diarrhœa. If the use of baths can be associated with the foregoing treatment, the child's improvement will be the more rapid. Great cleanliness and frequent change of clothes, careful ventilation of the child's chamber, and the wearing of a piece of fine flannel over the abdomen, are to be attended to.

2. In the chronic disorders of *adults,* in which Dr. L. employs milk and whey, the chief indication appears to be to stop the rapid emaciation. In most instances the origin of the disease was acute, and dated back months or even years, and its course was not clearly given. In general, the emaciation was attended with a slight febrile attack after the meal at noon or evening, and accompanied with more or less morning sweatings. In no case was suppuration of any viscus certain; in the majority of cases the certainty was quite the contrary. In a few cases, there was thirst after the morning perspirations, which ceased in the evening. Except in two cases there was no real hectic. The disposition was disturbed and particularly irritable, the sleep mostly interrupted, there was either anorexia or a sensation of pressure and obstruction in the stomach and bowels after taking food, or that which was eaten was again vomited. The bowels were constipated, and in females, previously regular in their menstrual function, this failed. An annoying dry cough occurred in some towards evening. Childbearing, menorrhagia, ague, and other imperfectly described female diseases, hypochondria and hysteria were among the diseases to which the origin of these complaints was ascribed. Many had to this time neglected themselves, others had adopted all sorts of tonic and stimulant treatment, all without benefit, or even harm. All these patients adopted an exclusively milk or whey treatment, with one exception, where, on account of the great feeling of hunger, something more was permitted.

*General Remarks on the Treatment itself.* (1.) Patients in the condition above described, subjected as they have been to various systems of treatment, will willingly submit to the milk-treatment, but it requires a discreet management on the part of the physician to ensure their observing it during eight or ten weeks, which will alone ensure a successful issue. (2.) In the choice between milk and whey, the degree of rapidity with which emaciation took place, the amount of appetite, the violence of the thirst, and the existence or non-existence of vomiting, were Dr. L.'s guides. Where the consumption was rapid, and where, even although emaciation was slow, there was little appetite and less thirst, and where the patient vomited what he had eaten, milk was always selected on account of its greater amount of nutritive matter. But if, when the emaciation advanced slowly, the appetite and thirst were very considerable, whey was preferred, of which the patients sometimes consumed three quarts in the twenty-four hours, a quantity which could not easily be digested of milk. When there were no particular grounds for preference, the choice of milk or whey was left to the patient himself. (3.) Often as Dr. L. has thus employed milk and whey, he has not in the circumstances mentioned met *with one contra-indication, nor in any individual case has he encountered any obstacle to the uninterrupted continuance of the treatment,* i. e. in the employment of an exclusively milk and whey treatment. Herein his experience leads him to different conclusions from those who speak of indications and counter-indications in the use of milk. (4.) With regard to the duration of the treatment, the author says that he did not prescribe a gradual transition to other diet, until he was fully convinced of the convalescence of his patient, that this rarely occurred before the expiration of eight weeks, and that in some cases it required a period of between four and five months.

[We have always been of opinion that the extremely active, or, as it is called, *heroic* treatment of chronic diseases, so prevalent in this country, is most injurious, and often fatal; and we have looked forward to much indirect benefit from the prevalence of the homœopathic doctrines, in demonstrating practically to the sectators of the *medicina proturbatrix*, that it is wiser and safer *to do nothing* when no rational indication presents itself, than to hazard the consequences of the blind application of potent drugs to a delicate or diseased system. In this point of view we regard the observations of Dr. Leviseur as important. Our own experience, now of many years, enables us to testify to the beneficial results of an analogous though not exactly similar mode of treatment in cases of the kind related; and we most confidently can promise our younger friends more success in numerous instances from adopting Dr. L.'s dietetic practice, than from the calomel and black-dose system which has so long disgraced British medicine.]

*Wochenschrift für die gesammte Heilkunde.* Nos. 25, 26. 1837.

---

### *On [Supposed] Inflammation of the Pancreas, observed during an Epidemic Parotitis.* By Dr. CONTI, of S. Natoglia.

IN October, 1835, the cold weather set in very suddenly and severely, so that the sides of the mountains and the vallies beneath were covered with snow; and from this time until May 1836, an epidemic parotitis made its appearance, that attacked a considerable number of persons of every age and sex. Besides the usual train of symptoms attendant upon the inflammation of the parotid glands, many of the males suffered from a similar affection of the testicles, while, in the females, the mammæ participated in the disease.

In the greater part, however, (above two hundred persons) no sooner was a feeling of uneasiness felt in the parotids, than a sharp oppressive pain came on in the epigastrium, attended with severe dyspnœa; attempts to vomit, with sour belchings and a flow of limpid water from the stomach; and followed by decided vomiting of a yellow frothy matter succeeded by a whitish fluid with a saline taste. At the same time the stomach was exquisitely tender to the touch, respiration was painful and laborious, and the patients complained of a sensation of weight and pressure, as though by a large stone, over the epigastric region, which was remarkably increased by swallowing even the smallest quantity of fluid. The vomitings were not copious but frequent, amounting to fifteen or twenty times in the course of the day. Some patients had hiccough; and the beating of the cæliac and aorta were perceptible to the eye. In some the epigastric pain extended itself over the whole abdomen, with a burning and pricking sensation of a rapid and fugacious character. An ordinary diarrhœa was soon followed by the discharge of a yellow frothy matter, and this by a white fluid equally frothy, like the white of egg beaten up, and emitting no fæcal odour. The patients who had diarrhœa complained less of the pain in the epigastrium; and although they suffered from the acid eructations and flow of limpid fluid, their attempts to vomit were ineffectual. The diarrhœa, like the vomiting, lasted six or seven days.

It is remarkable that the patients who suffered from this form of disease had no affection of the testicles or mammæ, while those who laboured under inflammation of those organs felt no uneasiness in the epigastrium.

The disease, when properly treated, did not last more than a fortnight; but, neglected or badly treated, it was often prolonged to six or nine weeks.

[The preceding narrative is curious, although the author adduces no positive proof of the seat of the disease being the pancreas, or of its inflammatory nature.]

*Bullettino delle Sc. Med. Jan.* 1837.

### *New Method for the Cure of Stammering.* By Dr. B. Voisin.

Dr. Voisin being afflicted with an impediment in his speech left no method untried, from the pebbles of Demosthenes to the methods of MM. Leich and Malbouche, for the purpose of removing it. The systems of M. Leich and his imitators instruct the stammerer to carry the tongue high, the point against the superior dental border of the incisors, so as to detach this organ as little as possible from the palate; and to observe a sort of measure in speaking. Dr. V. does not accord with these directions, and proposes another which he considers more simple and efficacious.

Chance first led him to the discovery of the method he recommends. He was reading a paper before a society, and wishing to do so with some energy, he happened to look in a mirror which was opposite him, and perceived that he rested the border of his right hand upon his chin, in a manner so as to depress the inferior maxilla and hold the mouth half open. The idea immediately suggested itself that this instinctive and mechanical movement might contribute to his reading more promptly and easily. In fact, upon ceasing the pressure, the difficulty of expression was quickly reproduced; but upon replacing his hand the freeness of articulation immediately returned. Endeavouring to give an account of this, he observed: 1st. That the mouth was kept half open, the distance between the teeth being a line or a line and a half. 2d. That the tongue, abandoned to itself, in the state of repose placed itself against the inferior dental border, whilst during pronunciation it is projected forwards and upwards, but is withdrawn almost immediately behind the alveolar arch. 3d. That a medium pressure is necessary upon the chin; this should be sufficiently strong to resist the muscles which move the inferior maxilla, without impeding its movement of elevation, so strong as to prevent perfect approximation. To produce this pressure and, at the same time, make it excusable, it is necessary to use a certain delicate art, so that the manœuvre may not appear forced, but on the contrary almost natural. This pressure should be made with the external border of the right or left hand indiscriminately, the thumb applied upon the chin and the fingers free. Since he has made the discovery he finds he frequently takes the position without thinking of it, and has observed the same in other individuals afflicted with impediment of speech. This habit does not appear to be peculiar to stammerers, since it is frequently assumed by timid persons when speaking in public. Dr. V. has only had an opportunity of trying it in two individuals, but the effect surpassed his expectations.

*Bulletin de l'Academie Royale de Médecine. September*, 1837.

